# Still They Come

**Published:** 1877-01

**Authors:** 


### STILL THEY COME.

Now that "Inhalation," for the cure of Consumption, has gone to the dogs, while its patronizers went to the grave, and when people have grown tired of guzzling villainous cod-liver oil by the tub-full; while *Naptha* and its "inventor" laid down in the grave together, after "a most brief" existence; and phosphate of lime, with its beautiful philosophic and microscopic theory, has long since hid its diminished head; and the great favorite of all, dram-drinking, has failed of its promised regeneration—*Cannabis Indica*, which, in vulgar English, is the hemp-plant boiled to a paste, then thinned with whisky, and sweetened with liquorice—it, too, has followed in the footsteps of its illustrious predecessors, notwithstanding its champion, "whose sands of life were almost run," had two barrels of that abundant article forwarded to him by "*express*," from New Orleans, for fear they might run out entirely, and prevent him from sending a "large bottle for two dollars" of the wonderful medicament to some afflicted son or daughter of Adam, who would have perished nineteen minutes and a quarter sooner than otherwise—said "*large bottle for two dollars*" costing, as we have ascertained by actual experiment, just thirty-one cents, bottle and all, even when the materials were of the freshest and purest that our next-door neighbor, *Tilden*, makes at his mammoth, celebrated laboratory; now that *Cannabis* has also failed of its promised wonder-workings, and is found to be most fitted for its old time use of going to seed and lint, and throttling the knaves who would divert it from its most legitimate use; a new aspirant for Consumptive celebrity has shot up into the British sky, and lays high claim to a deserved distinction above all that has gone before it—

### Hypophosphite of Lime and Iron.

The philosophy of it is, that it absorbs oxygen largely, and that its action is to dissolve tubercles, and get them speedily out of the system. How doctors and divines revel in magniloquence!

"Consumption is the oxydation of the exudation corpuscle," says the doctor.

"Anthropomorphism is theopneustic," responds the divine, in a New-York pulpit, in our hearing. The first means, in effect, that consumption is the gradual destruction of the lungs, which, perhaps, is known to most persons outside of an asylum; but as to Theopneusticability of Anthropomorphism, we beg leave to decline to define, for fear we might illustrate it into obscurity, and thus make it a *lucus a non lucendo*, distinguished only by the blackness of its darkness!

But let us retrograde to the beginning, take the back track to where we started from, and fetch up in plain, home-spun English.

The theory above admits that a consumptive person needs water, lime, phosphorus, and iron, and a plenty of oxygen in the largest quantities that Nature can take and convert to her uses. But there are two things which contain all these elements, combined by the greatest Chemist ever known—the Maker of all things—Beef and Pure Air.

The out-door air has all the oxygen in it that can possibly be used to advantage; for if one more proportion of oxygen is added to it, it becomes *aquafortis*. On the other hand, Beef, including all fish, flesh, and fowl, has full as much lime, and water, and phosphorus, and iron, as the system can appropriate; and this brings us back to the great truth, which we have not ceased to advocate these many years, that it is in vain we look to the prevention, arrest, and cure of consumptive disease, except in a vigorous digestion, and large, continuous, and moderate out-door activities.

Last month we quietly republished, without note or comment, an article which first appeared from our pen, *verbatim*, some years ago, one word only being changed, *Spirometry*, accented on the *second* syllable—it being easier of pronunciation than the original, which accented the fourth syllable. A writer in a British periodical advises the use of a spirometer, as curative of consumption—on the same principle precisely that we have recommended women patients especially, to use an india-rubber life-preserver, or to practice running up stairs with the mouth shut, or singing aloud while walking across the floor; and to male patients, to practice running with the mouth shut, with daily increasing distances; the effect of all being the same to induce instinctive efforts of deep inspirations in a natural way; while the same inspirations made artificially, if in too quick succession, induce vertigo, dizziness, and, possibly, apoplexy, or the rupture of a blood-vessel. This simple suggestion is worth millions of money to professional men, and whole ship-loads of physic to the common people, if it were applied with an intelligent discrimination.

The result of all these forms of exercise is one and the same; it lengthens the breath, makes way for a larger in-drawing and consequent use of the air we breathe, and the more we can take in, the more we can consume—the measure of one's radical improvement being an ability to run faster, and farther, and longer, with less fatigue, or to fully distend a life-preserver with fewer breaths.

Simple as these things are, they should be followed out, under the supervision of an intelligent physician, who must decide whether there be not some heart complication, making these exercises dangerous, or whether the strain put upon the lungs might not endanger the rupture of a blood-vessel—as the blindly-followed gymnastic exercises are known to occasion sometimes.

In addition to this, the physician must decide in each case how much exercise can be profitably taken; for if it go beyond a certain point—the actual fatigue line—that exercise has been destructive, instead of inuring to the building up of the system; which most important point, having been overlooked, has allowed many to exercise themselves to death.

Another point which imperatively requires an intelligent supervision, is the regulation of the food to its proper quantity and quality, and keeping the digestive function at the very highest point of healthful activity.

We are glad to have the names of quite a number of educated medical men on our subscription list—some of whom have added largely to that list, having the intelligence to perceive that the aim of our Journal is to throw the entire administration of medicine into the hands of educated men, and to banish self-medication from the land: and yet we could name a religious newspaper in this city, one of the largest of its class, which refused, without a reason, to advertise the "contents" of one of our numbers, and yet does not hesitate to notify its readers, for pay, where they can loan money for twenty-five per cent a year, where lottery book-stores may be found, and a common book may be had for a dollar, with, possibly, a ten-dollar note inside. Broad as creation is that phylactery!

On the other hand, a clergyman writes us, within a month: "You are aiming in the right direction, and cannot fail to make an impression for good; and the world will be the better for your having lived in it." Another, who does not live in a corner, says: "Every number of your Journal is an angel of mercy to divers households; and if I were rich, I would order five hundred copies a month for theological students."

But we digressed, while intending to say to our medical subscribers especially, that the suggestions in this article, in reference to the main principles of treatment of Consumption, are not exceeded in importance by anything that has been proposed in ancient or modern times; to wit, that the control of consumption is found only in the promotion of a vigorous digestion of food, and the large consumption of out-door air, in the employment of judicious muscular activities.

